# Circulating adipokine concentrations and risk of five obesity‐related cancers: A Mendelian randomization study

**DOI:** 10.1002/ijc.33338

**Published:** 2020-10-26

**Authors:** Niki L. Dimou, Nikos Papadimitriou, Daniela Mariosa, Mattias Johansson, Paul Brennan, Ulrike Peters, Stephen J. Chanock, Mark Purdue, D. Timothy Bishop, Manuela Gago‐Dominquez, Graham G. Giles, Victor Moreno, Elizabeth A. Platz, Catherine M. Tangen, Alicja Wolk, Wei Zheng, Xifeng Wu, Peter T. Campbell, Edward Giovannucci, Yi Lin, CCFR, Endometrial Cancer Association Consortium, Marc J. Gunter, Neil Murphy

**Affiliations:** ^1^ Section of Nutrition and Metabolism, International Agency for Research on Cancer Lyon France; ^2^ Section of Genetics, International Agency for Research on Cancer Lyon France; ^3^ Fred Hutchinson Cancer Research Center Seattle Washington USA; ^4^ Division of Cancer Epidemiology and Genetics, National Cancer Institute National Institutes of Health Bethesda Maryland USA; ^5^ Leeds Institute of Cancer and Pathology University of Leeds Leeds UK; ^6^ Fundación Gallega de Medicina Genómica, Grupo de Genéticadel Cáncer Instituto de Investigación Sanitaria de Santiago IDIS Complejo Hospitalario Univ. Santiago‐CHUS, SERGAS, Santiago de Compostela Spain; ^7^ Moores Cancer Center University of California San Diego La Jolla California USA; ^8^ Cancer Epidemiology Division Cancer Council Victoria Melbourne Victoria Australia; ^9^ Centre for Epidemiology and Biostatistics, Melbourne School of Population and Global Health The University of Melbourne Melbourne Victoria Australia; ^10^ Precision Medicine School of Clinical Sciences at Monash Health, Monash University Clayton Victoria Australia; ^11^ Oncology Data Analytics Program Catalan Institute of Oncology‐IDIBELL, L'Hospitalet de Llobregat Barcelona Spain; ^12^ CIBER Epidemiología y SaludPública (CIBERESP) Madrid Spain; ^13^ Department of Clinical Sciences, Faculty of Medicine University of Barcelona Barcelona Spain; ^14^ ONCOBEL Program, Bellvitge Biomedical Research Institute (IDIBELL), L'Hospitalet de Llobregat Barcelona Spain; ^15^ Department of Epidemiology, Johns Hopkins Bloomberg School of Public Health Baltimore Maryland USA; ^16^ SWOG Statistical Center, Fred Hutchinson Cancer Research Center Seattle Washington USA; ^17^ Institute of Environmental Medicine, Karolinska Institutet Stockholm Sweden; ^18^ Department of Surgical Sciences Uppsala University Uppsala Sweden; ^19^ Division of Epidemiology, Department of Medicine, Vanderbilt Epidemiology Center, Vanderbilt‐Ingram Cancer Center Vanderbilt University Nashville Tennessee USA; ^20^ Department of Epidemiology The University of Texas MD Anderson Cancer Center Houston Texas USA; ^21^ Department of Precision Health and Data Science, School of Public Health and the Second Affiliated Hospital Zhejiang University School of Medicine Hangzhou China; ^22^ Behavioral and Epidemiology Research Group, American Cancer Society Atlanta Georgia USA; ^23^ Department of Epidemiology, Harvard T.H. Chan School of Public Health Harvard University Boston Massachusetts USA; ^24^ Department of Nutrition T.H. Chan School of Public Health Boston Massachusetts USA; ^25^ Channing Division of Network Medicine, Brigham and Women's Hospital and Harvard Medical School Boston Massachusetts USA; ^26^ Public Health Sciences Division, Fred Hutchinson Cancer Research Center Seattle Washington USA

**Keywords:** adiponectin, cancer, leptin, Mendelian randomization, plasminogen activator inhibitor, soluble leptin receptor

## Abstract

Obesity is considered a chronic inflammatory state characterized by continued secretion of adipokines and cytokines. Experimental and epidemiological evidence indicates that circulating adipokines may be associated with the development of obesity‐related cancers, but it is unclear if these associations are causal or confounded. We examined potential causal associations of specific adipokines (adiponectin, leptin, soluble leptin receptor [sOB‐R] and plasminogen activator inhibitor‐1 [PAI‐1]) with five obesity‐related cancers (colorectal, pancreatic, renal cell carcinoma [RCC], ovarian and endometrial) using Mendelian randomization (MR) methods. We used summary**‐**level data from large genetic consortia for 114 530 cancer cases and 245 284 controls. We constructed genetic instruments using 18 genetic variants for adiponectin, 2 for leptin and 4 for both sOB‐R and PAI‐1 (*P* value for inclusion<5 × 10^−8^). Causal estimates were obtained using two‐sample MR methods. In the inverse‐variance weighted models, we found an inverse association between adiponectin and risk of colorectal cancer (odds ratio per 1 μg/mL increment in adiponectin concentration: 0.90 [95% confidence interval = 0.84‐0.97]; *P* = .01); but, evidence of horizontal pleiotropy was detected and the association was not present when this was taken into consideration. No association was found for adiponectin and risks of pancreatic cancer, RCC, ovarian cancer and endometrial cancer. Leptin, sOB‐R and PAI‐1 were also similarly unrelated to risk of obesity‐related cancers. Despite the large sample size, our MR analyses do not support causal effects of circulating adiponectin, leptin, sOB‐R and PAI‐1 concentrations on the development of five obesity‐related cancers.

## Abstract 2

**What's new?**

Chronic inflammation attributed to obesity may influence cancer development. However, little is known about the relationship between oncogenesis and changes in adipokine secretion stemming from immune cell infiltration in adipose tissue. Here, large‐scale Mendelian randomization analysis was used to assess possible causal associations of adipokine concentrations influenced by genetic variation and risk of five obesity‐related cancers, including renal cell carcinoma and colorectal, pancreatic, ovarian and endometrial cancer. In general, no association was detected between adipokines and the five malignancies, suggesting that adipokine levels have no causal influence on these cancers.

**Abbreviations**
BMI: body mass index
CCFR: Colon Cancer Family Registry
CI: confidence interval
CORECT: ColoRectal Transdisciplinary Study
EPIC: European Prospective Investigation into Cancer and Nutrition
GECCO: Genetics and Epidemiology of Colorectal Cancer
GWAS: genome‐wide association studies
HPFS: Health Professional Follow‐up
IVW: inverse‐variance weighted
LD: linkage disequilibrium
MR: Mendelian randomization
OR: odds ratio
PAI‐1: plasminogen activator inhibitor‐1
PCs: principal components
PPARG: Peroxisome Proliferator Activated Receptor Gamma
RCC: renal cell carcinoma
sOB‐R: soluble leptin receptor
WHI: Women's Health Initiative

## 1 INTRODUCTION

A substantial body of evidence has shown that excess adiposity is associated with a greater risk of developing many common cancers.^1^, ^2^ The biological pathways linking adiposity with cancer development are incompletely understood, but likely involve alterations in insulin signaling, sex hormone pathways and adipose tissue‐derived inflammation.^3^, ^4^ Obesity is considered as a chronic inflammatory state characterized by continued infiltration of adipose tissue by macrophages and other immune cells leading to increased or decreased adipose secretion of adipokines (such as adiponectin, leptin and plasminogen activator inhibitor‐1 [PAI‐1]) that may be linked to cancer development.

Adiponectin lowers secretion of inflammatory cytokines, improves insulin sensitivity and inhibits cell growth and angiogenesis, but is downregulated in obesity.^5^, ^6^ Multiple epidemiological studies have investigated the association between circulating adiponectin concentration and cancer risk with inverse relationships sometimes reported for endometrial, colorectal, renal cell carcinoma (RCC) and pancreatic cancer.^3^, ^7^, ^8^, ^9^, ^10^ Epidemiological studies that examined the association between circulating leptin concentration, which has pro‐inflammatory effects, and obesity‐related cancers have yielded inconsistent results.^9^, ^11^, ^12^ It is unclear if these mixed results were due to most studies failing to measure soluble leptin receptor (sOB‐R) concentrations, which may regulate the biological effects of circulating leptin concentration. Within the European Prospective Investigation into Cancer and Nutrition (EPIC) study, circulating concentration of sOB‐R was inversely associated with colorectal cancer, even after statistical adjustment for leptin concentrations, suggesting that sOB‐R may have an independent role in colorectal cancer development.^11^ It is currently unknown if sOB‐R is similarly associated with other obesity‐related cancers as these studies have not been conducted. In addition, few studies have examined the association between circulating PAI‐1 concentration (elevated in obesity) and cancer outcomes, although positive associations were found for colorectal cancer in the EPIC‐Italy and Women's Health Initiative (WHI) studies.^9^, ^13^

These previous observational epidemiological studies are vulnerable to residual confounding and reverse causality which make causal inference challenging. An alternative approach is Mendelian randomization (MR) that uses genetic variants robustly associated with the exposure of interest as instrumental variables to allow causal inference for the effect of an exposure on an outcome.^14^ MR analyses are largely free of conventional confounding and reverse causality as genetic variants are randomly assigned, and fixed, at conception.

We used a two‐sample MR framework to examine the associations of specific adipokines (ie, adiponectin, leptin, sOB‐R and PAI‐1) with five obesity‐related cancers using genetic variants associated with adipokines concentrations from published genome‐wide association studies (GWAS).^15^, ^16^, ^17^, ^18^, ^19^ We investigated the associations of these genetic variants with risks of colorectal cancer (58 221 cases and 67 694 controls), pancreatic cancer (7110 cases and 7264 controls), RCC (10 784 cases and 20 406 controls), ovarian cancer (25 509 cases and 40 941 controls) and endometrial cancer (12 906 cases and 108 979 controls).

## 2 METHODS

### 2.1 Adipokines data

We selected genetic variants for the MR analysis on the basis of a genome‐wide significant association with circulating adipokine concentrations (ie, *P* value threshold for inclusion at <5 × 10^−8^). For adiponectin, we used 18 variants in linkage disequilibrium (LD) below 1% (ie, rs2791552, rs2943641, rs2276853, rs3087866, rs13303, rs17366568, rs13133548, rs2925979, rs10282707, rs3735080, rs7134375, rs10861661, rs11057405 rs11057353, rs4311394, rs3865188, rs145119400, rs4805885) reported in a recent largest GWAS involving 67 739 individuals that were adjusted for age, sex, body mass index (BMI) and principal components (PCs) to account for possible population stratification.^20^ For leptin, two variants were incorporated (rs10487505 and rs6071166) adjusted for age^2^ BMI and PCs^17^ excluding rs780093 in the *GCKR* gene due to potential pleiotropy with several other traits (eg, urate concentrations, triglycerides, Crohn's disease, breast size).^21^, ^22^ We constructed an instrument for sOB‐R using four variants in the *LEPR* gene (rs17415296, rs4655537, rs17412403 and rs7535099), with low LD (*R* ^2^ ≤ 10%) to avoid underpowered MR analyses, that were adjusted for age, sex and BMI.^19^ In addition, we excluded rs3790438 yielding genome‐wide significance for sOB‐R since it was in almost perfect LD (ie, *R* ^2^ = 0.96) with rs17415296.^23^ Finally, performing LD pruning (*R* ^2^ ≤ 1%) resulted in four variants for PAI‐1 (rs11128603, rs2227631, rs6976053 and rs6486122) adjusted for age, sex and PCs.^16^ The variance explained in circulating adipokine concentration by the genetic instruments was 3%, 0.2%, 5% and 0.7% for adiponectin, leptin, sOB‐R and PAI‐1, respectively (Table S1).

### 2.2 Cancer data

Summary data for the associations of adipokine variants with colorectal cancer were obtained from a meta‐analysis of 125 915 participants (58 221 cases and 67 694 controls) within the ColoRectal Transdisciplinary Study (CORECT), the Colon Cancer Family Registry (CCFR) and the Genetics and Epidemiology of Colorectal Cancer (GECCO) consortia.^24^ GWAS data from pancreatic cancer samples (7110 cases and 7264 controls) were obtained from the PanScan and PanC4 consortia through the National Center for Biotechnology Information database of Genotypes and Phenotypes (dbGaP).^25^, ^26^, ^27^ Summary data for RCC (10 784 cases and 20 406 controls) were obtained from a recent GWAS.^28^ For ovarian cancer, summary data were obtained from a GWAS of 25 509 cases and 40 941 controls form the Ovarian Cancer Association Consortium (OCAC).^29^ For endometrial cancer, we obtained data from a GWAS of 12 906 cases and 108 979 controls from the Endometrial Cancer Association Consortium.^30^

### 2.3 Statistical power

Power calculations were performed based on the method suggested by Brion et al.^31^ We fixed the type‐I error rate at 0.05. Based on the aforementioned cancer case and control numbers, and assuming an *R* ^2^ of 3.0% (variance explained by the selected variants for circulating adiponectin), our study had 80% power to detect an odds ratio (OR) of 0.914/1.094 for colorectal cancer, 0.766/1.306 for pancreatic cancer, 0.831/1.204 for RCC, 0.881/1.135 for ovarian cancer and 0.870/1.150 for endometrial cancer. Power calculations by cancer subsite, subtype and by sex for various *R* ^2^ values are presented in Table 1.

**TABLE 1:** Number of cancer cases and controls and statistical power in Mendelian randomization study of adipokines and risk of cancer

Cancer type	Cases	Controls	Total	Proportion of cases	Minimum detectable odds ratio				
					*R* ^2^ = 0.01	*R* ^2^ = 0.02	*R* ^2^ = 0.03	*R* ^2^ = 0.04	*R* ^2^ = 0.05
*Colorectal cancer*									
Overall	58 221	67 694	125 915	0.46	0.855/1.170	0.894/1.118	0.914/1.094	0.924/1.082	0.932/1.073
Overall (men)	31 288	34 527	65 815	0.48	0.806/1.241	0.858/1.166	0.882/1.134	0.897/1.115	0.907/1.102
Overall (women)	26 843	32 820	59 663	0.45	0.797/1.255	0.850/1.176	0.876/1.141	0.892/1.121	0.903/1.108
Colon	31 083	67 694	98 777	0.31	0.831/1.203	0.873/1.145	0.897/1.115	0.910/1.099	0.918/1.089
Rectal	15 775	67 694	83 469	0.19	0.796/1.257	0.847/1.180	0.873/1.146	0.888/1.126	0.898/1.113
*Pancreatic cancer*									
Overall	7110	7264	14 374	0.49	0.632/1.582	0.722/1.386	0.766/1.306	0.793/1.261	0.831/1.230
Overall (men)	3861	4056	7917	0.49	0.542/1.845	0.646/1.549	0.696/1.437	0.733/1.365	0.757/1.321
Overall (women)	3252	3268	6520	0.50	0.508/1.968	0.617/1.621	0.672/1.489	0.709/1.410	0.735/1.360
*Renal cell carcinoma*									
Overall	10 784	20 406	31 190	0.35	0.730/1.369	0.798/1.253	0.831/1.204	0.851/1.175	0.865/1.156
*Ovarian*									
Overall	25 509	40 941	66 450	0.38	0.805/1.242	0.857/1.167	0.881/1.135	0.896/1.116	0.906/1.104
Serous	16 003	40 941	56 944	0.28	0.782/1.279	0.838/1.193	0.864/1.157	0.881/1.135	0.893/1.120
Clear‐cell	1366	40 941	42 307	0.03	0.554/1.806	0.638/1.568	0.683/1.464	0.714/1.401	0.736/1.358
Endometrioid	2810	40 941	43 751	0.06	0.635/1.575	0.712/1.405	0.752/1.329	0.778/1.285	0.797/1.254
*Endometrial cancer*									
Overall	12 906	108 979	121 885	0.11	0.792/1.262	0.845/1.184	0.870/1.150	0.885/1.130	0.896/1.116

*Note*: Minimum detectable odds ratio: assume 80% power, 5% alpha level and that 1% to 5% of adipokines heritability is explained by the variants used in this article.

### 2.4 Statistical analysis

We employed a fixed‐effects inverse‐variance weighted (IVW) MR method.^32^ For causal estimates from MR studies to be valid, three main assumptions must be satisfied: (a) the selected genetic variants used in the instrument are robustly associated with adipokine concentrations, (b) the genetic variants are not associated with any confounder of the adipokines and cancer relationship and (c) the genetic instrument should not affect the outcome independently of its effect on adipokine concentration. Assumption 1 was likely to be satisfied as only variants associated with adipokines at a genome‐wide significance level were used. For assumption 2, we acquired information for the association of the selected variants used in the instruments with other traits from the Phenoscanner.^33^ A series of statistical tests were performed to investigate the potential violation of MR assumption 3 and to assess the possible influence of horizontal pleiotropy on the causal estimates. We estimated the Cochran's *Q* statistic that quantifies the heterogeneity in effect sizes attributed to the selected genetic variants. When there was evidence for heterogeneity, we performed a random effects IVW approach in order to take into account this source of uncertainty.^34^ MR‐Egger regression was also used^35^ and the estimator from the weighted median approach.^36^ We conducted sensitivity analyses with variants associated with adiposity measures or insulin resistance excluded. We also restricted our analyses to *cis*‐acting variants. For adiponectin, we used rs17366568 variant in the *ADIPOQ* gene; for leptin, we used rs10487505 variant in the *LEP* gene; for PAI‐1, we used rs2227631 variant in the *SERPINE1* gene; while for sOB‐R, all four genetic variants (ie, rs17415296, rs4655537, rs17412403 and rs7535099) used are located in the *LEPR* gene.

For adiponectin and leptin, in sensitivity analyses, we also conducted analyses using selected variants unadjusted for BMI, to examine if collider bias may have influenced our results. In this scenario, we also accounted for pleiotropic effects acting via BMI using data from a recent GWAS of the GIANT consortium and the UK‐Biobank^37^ in a multivariable MR framework.^38^

## 3 RESULTS

### 3.1 Adiponectin

**FIGURE 1:**
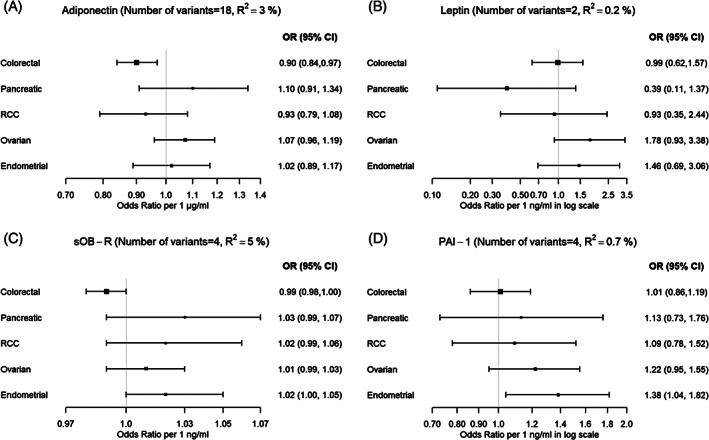
Mendelian randomization estimates between (A) adiponectin, (B) leptin, (C) soluble leptin receptor and (D) plasminogen activator inhibitor‐1 concentrations and cancer risk using the inverse‐variance weighted method. CI, confidence interval; OR, odds ratio; PAI‐1, plasminogen activator inhibitor‐1; RCC, renal cell carcinoma; sOB‐R, soluble leptin receptor

In the IVW models, we found an inverse association between adiponectin and risks of colorectal cancer (OR per 1 μg/mL increment in adiponectin concentrations: 0.90 (95% confidence interval [CI] = 0.84‐0.97); *P* = .01), with similar association found for men and women, colon cancer and rectal cancer (Figure 1 and Table S2). Near identical results were found when we used summary estimates of adiponectin unadjusted for BMI (Table S3). However, evidence of pleiotropy was detected and using robust MR methods (ie, MR‐Egger and Weighted median test) results were attenuated toward the null for all models except for colorectal cancer in women, for which weak evidence of an inverse effect was suggested by the MR‐Egger method (OR = 0.80 [95% CI = 0.62‐1.03]) (Table S2). The effect estimates for adiponectin and colorectal cancer were also slightly attenuated after the exclusion of variants associated with adiposity measures/insulin [overall colorectal cancer (OR = 0.92 [95% CI = 0.84‐1.01]; *P* = .09) (data not shown) or in a multivariable MR analysis accounting for BMI (overall colorectal cancer; OR = 0.92 [95% CI = 0.84‐1.01]; *P* = .1) (Table S3). Similar results were obtained when a *cis*‐acting variant was used as the genetic instrument (ie, rs17366568 in *ADIPOQ* gene) (overall colorectal cancer; OR = 0.92 [95% CI = 0.82‐1.03]; *P* = .16) (Table S2).

No association was found between adiponectin and pancreatic cancer (OR = 1.10 [95% CI = 0.91‐1.34]; *P* = .32), RCC (OR = 0.93 [95% CI = 0.79‐1.08]; *P* = .33), ovarian cancer (OR = 1.07 [95% CI = 0.96‐1.19]; *P* = .22) and endometrial cancer (OR = 1.02 [95% CI = 0.89‐1.17]; *P* = .75). Similar null results were found for the weighted median, MR‐Egger analyses, and when variants associated with obesity or insulin resistance were excluded from the instrument (Table S7).

### 3.2 Leptin and soluble leptin receptor

Leptin concentration was unrelated to risk of colorectal cancer (IVW OR per 1 ng/mL increase in log‐transformed leptin concentration: 0.99 [95% CI = 0.62‐1.57]; *P* = .96), pancreatic cancer (OR = 0.39 [95% CI = 0.11‐1.37]; *P* = .14), RCC (OR = 0.93 [95% CI = 0.35‐2.44]; *P* = .88), ovarian cancer (OR = 1.78 [95% CI = 0.93‐3.38]; *P* = .08) and endometrial cancer (OR = 1.46 [95% CI = 0.69‐3.06]; *P* = .32) (Figure 1 and Table S4). Similar results were found when analyses were restricted to the rs10487505 variant in the *LEP* gene (Table S4). No associations were also found for sOB‐R with any cancer type (Figure 1 and Table S5). For both leptin and sOB‐R, similar results were generally found by cancer subsite, subtype and sex, and no evidence of heterogeneity or pleiotropy was detected.

### 3.3 Plasminogen activator inhibitor‐1

PAI‐1 concentration was positively associated with endometrial cancer risk in the IVW models (OR = 1.38 [95% CI = 1.04‐1.82]; *P* = 0.03). This association was driven solely by the variant rs11128603 that is also associated with type‐2 diabetes, adiposity and body size traits; when this variant was excluded the association was attenuated toward the null (OR = 1.17 [95% CI = 0.86‐1.59]; *P* = .33). PAI‐1 concentration was not associated with risks of colorectal cancer (IVW OR per 1 ng/mL increase in log‐transformed PAI‐1 concentration: 1.01 [95% CI = 0.86‐1.19]; *P* = .92), pancreatic cancer (OR = 1.13 [95% CI = 0.73‐1.76]; *P* = .58), RCC (OR = 1.09 [95% CI = 0.78‐1.52]; *P* = 0.62) and ovarian cancer (OR = 1.22 [95% CI = 0.95‐1.55]; *P* = .11) (Figure 1 and Table S6). There was no association between genetically predicted circulating PAI‐1 concentration and any of the cancers when rs2227631 variant in the *SERPINE1* gene was used as the genetic instrument (Table S6). Similar results were generally found by cancer subsite, subtype and sex, and no evidence of heterogeneity or pleiotropy was detected.

## 4 DISCUSSION

In this large‐scale MR analysis, we found no evidence to support causal associations of genetically determined concentrations of adipokines and risk of five obesity‐related cancers. These results, together with the generally inconsistent observational epidemiological evidence, suggest that adiponectin, leptin, sOB‐R and PAI‐1 may not play a major role in the development of these five malignancies.

Adiponectin is predominantly secreted by visceral adipose tissue and is the most abundant adipokine, with circulating concentrations inversely correlated with adiposity. Numerous epidemiological studies have investigated the circulating adiponectin and cancer relationship, with inverse associations generally observed.^8^, ^30^ For colorectal cancer, higher circulating adiponectin concentrations have been associated with lower risk within the EPIC, Women's Health Initiative (WHI) and Health Professional Follow‐up (HPFS) studies, with these associations usually attenuated after BMI adjustment.^7^, ^9^, ^10^ In our MR analysis, we found an inverse association between genetically determined adiponectin concentration and colorectal cancer, which was not robust in the MR‐Egger and Weighted median approach, which suggests that horizontal pleiotropy may have influenced this result. Additionally, the inverse association was attenuated toward the null after removing variants that were associated with adiposity. Collectively, these results do not support adiponectin having a causal effect on colorectal cancer risk.

For the other cancers, our results indicate that circulating adiponectin is unlikely to be causally related to tumorigenesis. Epidemiological studies examining the adiponectin and pancreatic risk association have reported mixed results. A nested case‐control study of five cohorts in the United States reported that higher circulating adiponectin concentrations were associated with lower pancreatic cancer risk (OR for highest vs compared with lowest quintile 0.66 [95% CI = 0.44‐0.97]),^39^ whereas a null result was found between adiponectin and pancreatic cancer in an analysis of the EPIC study.^40^ The evidence of circulating adiponectin concentration and risks of RCC and endometrial cancer from prospective epidemiological studies is mixed, with inverse associations reported by some^41^, ^42^, ^43^ studies, but not by others.^44^, ^45^ It is possible that this inconsistency in results is a consequence of measurement error, residual confounding and reverse causality inherent to observational epidemiology influencing these analyses to varying degrees. In contrast, our MR analyses should be largely free of conventional confounding and reverse causality which allows causal inference.

Our MR analyses for colorectal cancer and all other obesity‐related cancers considered found no associations with genetically determined leptin and sOB‐R concentrations. Multiple epidemiological studies have investigated the association between circulating leptin and risks of obesity‐related cancers. Circulating concentrations of leptin were positively associated with colon cancer risk in a Norwegian nested case‐control study (OR comparing the highest vs the lowest quartile 2.72 [95% CI = 1.44‐5.12]).^12^ Similarly, in a WHI case‐cohort study, a positive association between serum leptin concentration and colorectal cancer risk was found, even after adjustment for insulin concentrations.^9^ An analysis in the EPIC study found no association for circulating leptin, but did observe an inverse association for sOB‐R with colorectal cancer.^11^ The null results we found for leptin and sOB‐R are largely consistent with previous epidemiological evidence for pancreatic cancer,^46^ but inconsistent for RCC and endometrial cancer, for which the few prospective studies conducted have generally found positive associations for circulating leptin concentration.^43^, ^47^

We found no association between genetically determined PAI‐I concentration and risks of colorectal, pancreatic, renal cell and ovarian cancer. Few epidemiological studies have investigated the relationship between circulating PAI‐1 concentration and obesity‐related cancers. For colorectal cancer, analyses in the EPIC‐Italy and WHI studies found positive association for circulating PAI‐1,^9^, ^13^ although the WHI positive association were attenuated toward the null after adjusting for circulating insulin concentration.^9^ Few epidemiological studies have examined the association of PAI‐1 with risks of pancreatic, RCC, ovarian cancer and endometrial cancer. Limited data exist for PAI‐1 and RCC, with some evidence of an association between PAI‐1 with angiogenesis of tumors in clear cell RCC.^48^ For endometrial cancer, our MR analysis yielded a positive association with PAI‐I. However, this association was driven solely by one variant (rs11128603) in the Peroxisome Proliferator Activated Receptor Gamma (*PPARG*) gene, which is also associated with type‐2 diabetes, adiposity and body size traits.

A large body of epidemiological research has investigated the relationships between circulating concentrations of adipokines and cancer development, with inconsistent results found. We conducted the largest and most comprehensive MR study investigating potential causal associations between genetically determined circulating adipokines concentrations and risks of five obesity‐related cancers. Our results are consistent with the findings of a recent MR study that showed no association between adiponectin, sOB‐R and PAI‐1 concentrations and breast cancer risk.^49^ Though it is not possible to prove the validity of some of the MR assumptions with summarized data, we performed various sensitivity analyses and investigated potential associations with secondary phenotypes of interest, including BMI and insulin resistance. Importantly, our results were similar when we restricted the genetic instruments to include *cis*‐acting variants, suggesting that pleiotropy did not markedly influence our findings. Power calculations indicated that our analyses were adequately powered to detect effect sizes comparable with prior observational studies that reported associations between adipokine concentrations and these cancers,^7^, ^11^, ^12^, ^13^, ^43^, ^47^ with the exception of some of the pancreatic cancer and cancer subtype models for which GWAS case‐control numbers were relatively low.^39^ A further limitation was that the GWAS for adipokine concentrations included largely middle‐aged individuals; therefore, it is unknown to what extent these data capture early life exposures which may be of relevance for the development of these cancers. In addition, the summary level data that we used did not allow for stratified analyses by covariates of interest, such as menopausal status, circulating insulin concentration, family history of cancer, physical activity, smoking and alcohol. Further, for sOB‐R and PAI‐1, sex‐specific GWAS estimates were unavailable so we used the sex‐combined estimates for all analyses. The data that we retrieved for adiponectin, leptin and sOB‐R were adjusted for BMI and this may have introduced collider bias into these analyses; however, in the GWAS for adiponectin, it was estimated that any bias resulting from adjusting for BMI was minimal.^20^ This was further corroborated by our sensitivity analysis using the estimates unadjusted for BMI for adiponectin and leptin that resulted in similar effect estimates. Finally, results from a recent empirical study suggest that using covariate adjusted GWAS summary estimates is unlikely to markedly influence MR effect estimates.^50^

In summary, using a MR analytical framework, our results do not support causal effects of circulating adipokines on risk of five obesity‐related cancers. Although we cannot rule out the existence of weak associations for specific cancer subtypes for which our analyses were possibly underpowered, or undetected violations to the MR assumptions for causal inference, our results suggest that adiponectin, leptin, sOB‐R and PAI‐1 do not play a causal role in cancer development.

## FUNDING INFORMATION

Genetics and Epidemiology of Colorectal Cancer Consortium (GECCO): National Cancer Institute, National Institutes of Health, U.S. Department of Health and Human Services (U01 CA164930, U01 CA137088, R01 CA059045, U01 CA164930, R21 CA191312).

ASTERISK: a Hospital Clinical Research Program (PHRC‐BRD09/C) from the University Hospital Center of Nantes (CHU de Nantes) and supported by the Regional Council of Pays de la Loire, the Groupement des Entreprises Françaisesdans la Luttecontre le Cancer (GEFLUC), the Association Anne de Bretagne Génétique and the Ligue Régionale Contre le Cancer (LRCC).

The ATBC Study is supported by the Intramural Research Program of the U.S. National Cancer Institute, National Institutes of Health, and by U.S. Public Health Service contract HHSN261201500005C from the National Cancer Institute, Department of Health and Human Services.

CLUE II: This research was funded by the American Institute for Cancer Research and the Maryland Cigarette Restitution Fund at Johns Hopkins, and the NCI (P30 CA006973 to W.G. Nelson).

COLO2&3: National Institutes of Health (R01 CA60987).

ColoCare: This work was supported by the National Institutes of Health (grant numbers R01 CA189184 (Li/Ulrich), U01 CA206110 (Ulrich/Li/Siegel/Figueireido/Colditz, 2P30CA015704‐40 (Gilliland), R01 CA207371 (Ulrich/Li)), the Matthias Lackas‐Foundation, the German Consortium for Translational Cancer Research, and the EU TRANSCAN initiative.

The Colon Cancer Family Registry (CCFR, www.coloncfr.org) was supported in part by funding from the National Cancer Institute (NCI), National Institutes of Health (NIH) (award U01 CA167551) and through U01/U24 cooperative agreements from NCI with the following CCFR centers: Australasian (CA074778 and CA097735), Ontario (OFCCR) (CA074783), Seattle (SFCCR) (CA074794 [and R01 CA076366 to PAN]), USC Consortium (CA074799), Mayo Clinic (CA074800), and Hawaii (CA074806). Support for case ascertainment was provided in part from the Surveillance, Epidemiology, and End Results (SEER) Program and the following U.S. state cancer registries: AZ, CO, MN, NC, NH; and by the Victoria Cancer Registry (Australia) and Ontario Cancer Registry (Canada). Additional funding for the OFCCR/ARCTIC wasthrough award GL201‐043 from the Ontario Research Fund (to BWZ), award 112746 from the Canadian Institutes of Health Research (to TJH), through a Cancer Risk Evaluation (CaRE) Program grant from the Canadian Cancer Society (to SG), and through generous support from the Ontario Ministry of Research and Innovation. The SCCFR Illumina HumanCytoSNP array was supported through NCI award R01 CA076366 (to PAN).The CCFR Set‐1 (Illumina 1 M/1 M‐Duo) and Set‐2 (Illumina Omni1‐Quad) scans were supported by NIH awards U01 CA122839 and R01 CA143247 (to GC). The CCFR Set‐3 (Affymetrix Axiom CORECT Set array) was supported by NIH award U19 CA148107 and R01 CA81488 (to SBG). The CCFR Set‐4 (Illumina OncoArray 600 K SNP array**)** was supported by NIH award U19 CA148107 (to SBG) and by the Center for Inherited Disease Research (CIDR), which is funded by the NIH to the Johns Hopkins University, contract number HHSN268201200008I. Colon Cancer Family Registry (CCFR): The content of this manuscript does not necessarily reflect the views or policies of the NIH or any of the collaborating centers in the CCFR, nor does mention of trade names, commercial products, or organizations imply endorsement by the US Government, any cancer registry, or the CCFR.

COLON: The COLON study is sponsored by WereldKankerOnderzoekFonds, including funds from grant 2014/1179 as part of the World Cancer Research Fund International Regular Grant Programme, by Alped'Huzes and the Dutch Cancer Society (UM 2012‐5653, UW 2013‐5927, UW2015‐7946), and by TRANSCAN (JTC2012‐MetaboCCC, JTC2013‐FOCUS). The Nqplus study is sponsored by a ZonMW investment grant (98‐10030); by PREVIEW, the project PREVention of diabetes through lifestyle intervention and population studies in Europe and around the World (PREVIEW) project which received funding from the European Union Seventh Framework Programme (FP7/2007‐2013) under grant no. 312057; by funds from TI Food and Nutrition (cardiovascular health theme), a public‐private partnership on precompetitive research in food and nutrition; and by FOODBALL, the Food Biomarker Alliance, a project from JPI Healthy Diet for a Healthy Life.

Colorectal Cancer Transdisciplinary (CORECT) Study: The CORECT Study was supported by the National Cancer Institute, National Institutes of Health (NCI/NIH), U.S. Department of Health and Human Services (grant numbers U19 CA148107, R01 CA81488, P30 CA014089, R01 CA197350,; P01 CA196569; R01 CA201407) and National Institutes of Environmental Health Sciences, National Institutes of Health (grant number T32 ES013678).

CORSA: “ÖsterreichischeNationalbankJubiläumsfondsprojekt” (12511) and Austrian Research Funding Agency (FFG) grant 829675.

CPS‐II: The American Cancer Society funds the creation, maintenance, and updating of the Cancer Prevention Study‐II (CPS‐II) cohort. This study was conducted with Institutional Review Board approval.

CRCGEN: Colorectal Cancer Genetics & Genomics, Spanish study was supported by Instituto de Salud Carlos III, co‐funded by FEDER funds ‐a way to build Europe‐ (grants PI14‐613 and PI09‐1286), Agency for Management of University and Research Grants (AGAUR) of the Catalan Government (grant 2017SGR723), and Junta de Castilla y León (grant LE22A10‐2). Sample collection of this work was supported by the Xarxa de Bancs de Tumors de Catalunya sponsored by Pla Director d'Oncología de Catalunya (XBTC), PlataformaBiobancos PT13/0010/0013 and ICOBIOBANC, sponsored by the Catalan Institute of Oncology.

Czech Republic CCS: This work was supported by the Grant Agency of the Czech Republic (grants CZ GA CR: GAP304/10/1286 and 1585) and by the Grant Agency of the Ministry of Health of the Czech Republic (grants AZV 15‐27580A and AZV 17‐30920A).

DACHS: This work was supported by the German Research Council (BR 1704/6‐1, BR 1704/6‐3, BR 1704/6‐4, CH 117/1‐1, HO 5117/2‐1, HE 5998/2‐1, KL 2354/3‐1, RO 2270/8‐1 and BR 1704/17‐1), the Interdisciplinary Research Program of the National Center for Tumor Diseases (NCT), Germany, and the German Federal Ministry of Education and Research (01KH0404, 01ER0814, 01ER0815, 01ER1505A and 01ER1505B).

DALS: National Institutes of Health (R01 CA48998 to M. L. Slattery).

EDRN: This work is funded and supported by the NCI, EDRN Grant (U01 CA 84968‐06).

EPIC: The coordination of EPIC is financially supported by the European Commission (DGSANCO) and the International Agency for Research on Cancer. The national cohorts are supported by Danish Cancer Society (Denmark); LigueContre le Cancer, Institut Gustave Roussy, MutuelleGénérale de l'EducationNationale, Institut National de la Santé et de la RechercheMédicale (INSERM) (France); German Cancer Aid, German Cancer Research Center (DKFZ), Federal Ministry of Education and Research (BMBF), Deutsche Krebshilfe, DeutschesKrebsforschungszentrum and Federal Ministry of Education and Research (Germany); the Hellenic Health Foundation (Greece); Associazione Italiana per la RicercasulCancro‐AIRCItaly and National Research Council (Italy); Dutch Ministry of Public Health, Welfare and Sports (VWS), Netherlands Cancer Registry (NKR), LK Research Funds, Dutch Prevention Funds, Dutch ZON (ZorgOnderzoek Nederland), World Cancer Research Fund (WCRF), Statistics Netherlands (The Netherlands); ERC‐2009‐AdG 232997 and Nordforsk, Nordic Centre of Excellence programme on Food, Nutrition and Health (Norway); Health Research Fund (FIS), PI13/00061 to Granada, PI13/01162 to EPIC‐Murcia, Regional Governments of Andalucía, Asturias, Basque Country, Murcia and Navarra, ISCIII RETIC (RD06/0020) (Spain); Swedish Cancer Society, Swedish Research Council and County Councils of Skåne and Västerbotten (Sweden); Cancer Research UK (14136 to EPIC‐Norfolk; C570/A16491 and C8221/A19170 to EPIC‐Oxford), Medical Research Council (1000143 to EPIC‐Norfolk, MR/M012190/1 to EPICOxford) (United Kingdom).

EPICOLON: This work was supported by grants from Fondo de Investigación Sanitaria/FEDER (PI08/0024, PI08/1276, PS09/02368, P111/00219, PI11/00681, PI14/00173, PI14/00230, PI17/00509, 17/00878, Acción Transversal de Cáncer), Xunta de Galicia (PGIDIT07PXIB9101209PR), Ministerio de Economia y Competitividad (SAF07‐64873, SAF 2010‐19273, SAF2014‐54453R), Fundación Científica de la Asociación Española contra el Cáncer (GCB13131592CAST), Beca Grupo de Trabajo “Oncología” AEG (Asociación Española de Gastroenterología), Fundación Privada Olga Torres, FP7 CHIBCHA Consortium, Agència de Gestiód'Ajuts Universitarisi de Recerca (AGAUR, Generalitat de Catalunya, 2014SGR135, 2014SGR255, 2017SGR21, 2017SGR653), Catalan Tumor Bank Network (Pla Director d'Oncologia, Generalitat de Catalunya), PERIS (SLT002/16/00398, Generalitat de Catalunya), CERCA Programme (Generalitat de Catalunya) and COST Action BM1206 and CA17118. CIBERehd is funded by the Instituto de Salud Carlos III.

ESTHER/VERDI. This work was supported by grants from the Baden‐Württemberg Ministry of Science, Research and Arts and the German Cancer Aid.

Harvard cohorts (HPFS, NHS, PHS): HPFS is supported by the National Institutes of Health (P01 CA055075, UM1 CA167552, U01 CA167552, R01 CA137178, R01 CA151993, R35 CA197735, K07 CA190673, and P50 CA127003), NHS by the National Institutes of Health (R01 CA137178, P01 CA087969, UM1 CA186107, R01 CA151993, R35 CA197735, K07CA190673, and P50 CA127003) and PHS by the National Institutes of Health (R01 CA042182).

Hawaii Adenoma Study: NCI grants R01 CA72520.

HCES‐CRC: the Hwasun Cancer Epidemiology Study‐Colon and Rectum Cancer (HCES‐CRC; grants from Chonnam National University Hwasun Hospital, HCRI15011‐1).

Kentucky: This work was supported by the following grant support: Clinical Investigator Award from Damon Runyon Cancer Research Foundation (CI‐8); NCI R01CA136726.

LCCS: The Leeds Colorectal Cancer Study was funded by the Food Standards Agency and Cancer Research UK Programme Award (C588/A19167).

MCCS cohort recruitment was funded by VicHealth and Cancer Council Victoria. The MCCS was further supported by Australian NHMRC grants 509348, 209057, 251553 and 504711 and by infrastructure provided by Cancer Council Victoria. Cases and their vital status were ascertained through the Victorian Cancer Registry (VCR) and the Australian Institute of Health and Welfare (AIHW), including the National Death Index and the Australian Cancer Database.

MEC: National Institutes of Health (R37 CA54281, P01 CA033619, and R01 CA063464).

MECC: This work was supported by the National Institutes of Health, U.S. Department of Health and Human Services (R01 CA81488 to SBG and GR).

MSKCC: The work at Sloan Kettering in New York was supported by the Robert and Kate Niehaus Center for Inherited Cancer Genomics and the Romeo Milio Foundation. Moffitt: This work was supported by funding from the National Institutes of Health (grant numbers R01 CA189184, P30 CA076292), Florida Department of Health Bankhead‐Coley Grant 09BN‐13, and the University of South Florida Oehler Foundation. Moffitt contributions were supported in part by the Total Cancer Care Initiative, Collaborative Data Services Core, and Tissue Core at the H. Lee Moffitt Cancer Center & Research Institute, a National Cancer Institute‐designated Comprehensive Cancer Center (grant number P30 CA076292).

NCCCS I & II: We acknowledge funding support for this project from the National Institutes of Health, R01 CA66635 and P30 DK034987.

NFCCR: This work was supported by an Interdisciplinary Health Research Team award from the Canadian Institutes of Health Research (CRT 43821); the National Institutes of Health, U.S. Department of Health and Human Serivces (U01 CA74783); and National Cancer Institute of Canada grants (18223 and 18226). The authors wish to acknowledge the contribution of Alexandre Belisle and the genotyping team of the McGill University and Génome Québec Innovation Centre, Montréal, Canada, for genotyping the Sequenom panel in the NFCCR samples. Funding was provided to Michael O. Woods by the Canadian Cancer Society Research Institute.

NSHDS: Swedish Cancer Society; Cancer Research Foundation in Northern Sweden; Swedish Research Council; J C Kempe Memorial Fund; Faculty of Medicine, Umeå University, Umeå, Sweden; and Cutting‐Edge Research Grant from the County Council of Västerbotten, Sweden.

OFCCR: The Ontario Familial Colorectal Cancer Registry was supported in part by the National Cancer Institute (NCI) of the National Institutes of Health (NIH) under award U01 CA167551 and award U01/U24 CA074783 (to SG). Additional funding for the OFCCR and ARCTIC testing and genetic analysis was through and a Canadian Cancer Society CaRE (Cancer Risk Evaluation) program grant and Ontario Research Fund award GL201‐043 (to BWZ), through the Canadian Institutes of Health Research award 112746 (to TJH), and through generous support from the Ontario Ministry of Research and Innovation.

OSUMC: OCCPI funding was provided by Pelotonia and HNPCC funding was provided by the NCI (CA16058 and CA67941).

PLCO: Intramural Research Program of the Division of Cancer Epidemiology and Genetics and supported by contracts from the Division of Cancer Prevention, National Cancer Institute, NIH, DHHS. Funding was provided by National Institutes of Health (NIH), Genes, Environment and Health Initiative (GEI) Z01 CP 010200, NIH U01 HG004446, and NIH GEI U01 HG 004438.

SCCFR: The Seattle Colon Cancer Family Registry was supported in part by the National Cancer Institute (NCI) of the National Institutes of Health (NIH) under awards U01 CA167551. Additional support for the SFCCR, Postmenopausal Hormones and Colon Cancer (PMH) study and the SCCFR Illumina HumanCytoSNP array were through NCI/NIH awards U01/U24 CA074794 and R01 CA076366 (to PAN).

SEARCH: The University of Cambridge has received salary support in respect of PDPP from the NHS in the East of England through the Clinical Academic Reserve. Cancer Research UK (C490/A16561); the UK National Institute for Health Research Biomedical Research Centres at the University of Cambridge.

SELECT: Research reported in this publication was supported in part by the National Cancer Institute of the National Institutes of Health under Award Numbers U10 CA37429 (CD Blanke), and UM1 CA182883 (CM Tangen/IM Thompson). The content is solely the responsibility of the authors and does not necessarily represent the official views of the National Institutes of Health.

SMS: This work was supported by the National Cancer Institute (grant P01 CA074184 to J.D.P. and P.A.N., grants R01 CA097325, R03 CA153323, and K05 CA152715 to P.A.N., and the National Center for Advancing Translational Sciences at the National Institutes of Health (grant KL2 TR000421 to A.N.B.‐H.)

The Swedish Low‐risk Colorectal Cancer Study: The study was supported by grants from the Swedish research council; K2015‐55X‐22674‐01‐4, K2008‐55X‐20157‐03‐3, K2006‐72X‐20157‐01‐2 and the Stockholm County Council (ALF project).

Swedish Mammography Cohort and Cohort of Swedish Men: This work is supported by the Swedish Research Council /Infrastructure grant, the Swedish Cancer Foundation, and the Karolinska Institute´s Distinguished Professor Award to AlicjaWolk.

UK Biobank: This research has been conducted using the UK Biobank Resource under Application Number 8614.

VITAL: National Institutes of Health (K05 CA154337).

Women's Health Initiative: The WHI program is funded by the National Heart, Lung, and Blood Institute, National Institutes of Health, U.S. Department of Health and Human Services through contracts HHSN268201100046C, HHSN268201100001C, HHSN268201100002C, HHSN268201100003C, HHSN268201100004C, and HHSN271201100004C.

This research was supported in part by the Intramural Research Program of the NIH and the National Cancer Institute.

## CONFLICT OF INTEREST

The authors declare no potential conflict of interest.

## DISCLAIMER

Where authors are identified as personnel of the International Agency for Research on Cancer/World Health Organization, the authors alone are responsible for the views expressed in this article and they do not necessarily represent the decisions, policy or views of the International Agency for Research on Cancer/World Health Organization. The content is solely the responsibility of the authors and does not necessarily represent the official views of the National Institutes of Health.

## Supporting information


**Appendix S1**: Supporting Information

## Data Availability

The data that support the findings of this study are available from the corresponding author upon reasonable request.
