# Supplementary material for: Circulating adipokine concentrations and risk of five obesity‐related cancers: A Mendelian randomization study
Source: Int J Cancer. 2020 Oct 26;148(7):1625–36. doi: 10.1002/ijc.33338 (PMC7894468; doi:10.1002/ijc.33338)
Supplement: Supplementary file 1 — Appendix S1: Supporting Information [file IJC-148-1625-s001.pdf]

Circulating adipokine concentrations and risk of five obesity-related cancers: a Mendelian randomization study

Niki L Dimou[1], Nikos Papadimitriou[1], Daniela Mariosa[2], Mattias Johansson[2], Paul Brennan[2], Ulrike Peters[3], Stephen J Chanock[4], Mark Purdue[4], D Timothy Bishop[5], Manuela Gago-Dominquez[6, 7],Graham G Giles[8, 9, 10], Victor Moreno[11, 12, 13, 14], Elizabeth A Platz[15], Catherine M Tangen[16], Alicja Wolk[17, 18], Wei Zheng[19], Xifeng Wu[20, 21], Peter T Campbell[22], Edward Giovannucci[23, 24, 25], Yi Lin[26], CCFR, Endometrial Cancer Association Consortium, Marc J Gunter[1], Neil Murphy[1]

Table of contents

Supplementary Tables

Supplementary Table 1 p. 2-3

Supplementary Table 2 p. 4-7

Supplementary Table 3 p. 8-9

Supplementary Table 4 p. 10-11

Supplementary Table 5 p. 12-14

Supplementary Table 6 p. 15-18

Supplementary Table 7 p. 19-25

Supplementary Table 1. Characteristics of genetic variants associated with adipokines in published GWAS.

Adipokine	Study (Pubmed ID)	SNPs	Effect allele	Beta	SE	P-value	R^2
adiponectin	Spracklen, C.N., et al. (31178129)	rs2791552	C	-0.054	0.007	1.75E-14	3.0%
adiponectin	Spracklen, C.N., et al. (31178129)	rs2943641	C	-0.060	0.006	1.76E-21	
adiponectin	Spracklen, C.N., et al. (31178129)	rs2276853	A	-0.033	0.006	3.35E-08	
adiponectin	Spracklen, C.N., et al. (31178129)	rs3087866	C	-0.040	0.007	2.14E-08	
adiponectin	Spracklen, C.N., et al. (31178129)	rs13303	C	0.060	0.006	2.69E-21	
adiponectin	Spracklen, C.N., et al. (31178129)	rs17366568	A	-0.228	0.009	1.43E-145	
adiponectin	Spracklen, C.N., et al. (31178129)	rs13133548	A	-0.039	0.006	5.91E-12	
adiponectin	Spracklen, C.N., et al. (31178129)	rs2925979	C	0.082	0.006	1.38E-37	
adiponectin	Spracklen, C.N., et al. (31178129)	rs10282707	T	-0.040	0.006	1.96E-10	
adiponectin	Spracklen, C.N., et al. (31178129)	rs3735080	T	-0.049	0.007	7.70E-13	
adiponectin	Spracklen, C.N., et al. (31178129)	rs7134375	A	0.054	0.006	3.69E-20	
adiponectin	Spracklen, C.N., et al. (31178129)	rs10861661	C	-0.040	0.007	1.75E-09	
adiponectin	Spracklen, C.N., et al. (31178129)	rs11057405	A	-0.080	0.011	2.90E-14	
adiponectin	Spracklen, C.N., et al. (31178129)	rs11057353	C	0.054	0.006	2.74E-19	
adiponectin	Spracklen, C.N., et al. (31178129)	rs4311394	G	-0.045	0.007	1.68E-10	
adiponectin	Spracklen, C.N., et al. (31178129)	rs3865188	T	-0.035	0.006	1.14E-08	
adiponectin	Spracklen, C.N., et al. (31178129)	rs145119400	A	-0.300	0.042	8.95E-13	
adiponectin	Spracklen, C.N., et al. (31178129)	rs4805885	C	0.058	0.006	2.85E-25	
leptin	Kilpelainen, T.O., et al. (26833098)	rs10487505	G	0.029	0.004	1.99E-12	0.2%
leptin	Kilpelainen, T.O., et al. (26833098)	rs6071166	C	0.024	0.004	1.75E-08	
leptin receptor	Suhre, K., et al. (28240269)	rs17415296	C	1.400	0.033	4.47E-229	5.0%
leptin receptor	Suhre, K., et al. (28240269)	rs4655537	A	0.350	0.044	7.42E-15	
leptin receptor	Suhre, K., et al. (28240269)	rs17412403	T	0.328	0.043	8.66E-14	
leptin receptor	Suhre, K., et al. (28240269)	rs7535099	G	0.346	0.051	3.06E-11	

plasminogen activator inhibitor-1	Huang, J., et al. (22990020)	rs11128603	A	0.066	0.012	9.4E-08	
plasminogen activator inhibitor-1	Huang, J., et al. (22990020)	rs2227631	A	0.073	0.007	3.20E-24	0.7%
plasminogen activator inhibitor-1	Huang, J., et al. (22990020)	rs6976053	T	0.048	0.007	5.80E-13	
plasminogen activator inhibitor-1	Huang, J., et al. (22990020)	rs6486122	T	0.046	0.0072	1.7E-10	

GWAS, genome-wide association studies; SNPs, single nucleotide polymorphisms

Supplementary Table 2. Mendelian randomization estimates between adiponectin concentration and cancer risk.

Cancer type	Methods	Estimates (OR)	95% CI	P-value	P-value for pleiotropy or heterogeneity
**Colorectal**					
**Overall**	Inverse-variance weighted	0.90	(0.84-0.97)	0.01	0.01
	Inverse-variance weighted (random effects)	0.90	(0.82-1.00)	0.04	0.01
	Wald (rs17366568)	0.92	(0.82-1.03)	0.16	NA
	MR-Egger	0.98	(0.79-1.21)	0.82	0.37
	Weighted median	0.92	(0.83-1.02)	0.11	NA
**Overall (men)**	Inverse-variance weighted	0.89	(0.80-0.99)	0.04	0.02
	Inverse-variance weighted (random effects)	0.89	(0.79-1.04)	0.16	0.02
	Wald (rs17366568)	0.96	(0.82-1.12)	0.57	NA
	MR-Egger	1.01	(0.70-1.45)	0.96	0.39
	Weighted median	0.95	(0.83-1.10)	0.51	NA
**Overall (women)**	Inverse-variance weighted	0.90	(0.81-1.01)	0.07	0.63
	Wald (rs17366568)	0.88	(0.74-1.06)	0.17	NA
	MR-Egger	0.80	(0.62-1.03)	0.08	0.29
	Weighted median	0.88	(0.76-1.02)	0.10	NA
**Colon**	Inverse-variance weighted	0.90	(0.83-0.98)	0.02	0.09
	Wald (rs17366568)	0.94	(0.82-1.09)	0.41	NA
	MR-Egger	0.97	(0.78-1.22)	0.81	0.41
	Weighted median	0.93	(0.83-1.06)	0.28	NA
**Rectal**	Inverse-variance weighted	0.91	(0.82-1.02)	0.11	0.06

	Wald (rs17366568)	0.96	(0.80-1.16)	0.70	NA
	MR-Egger	1.11	(0.83-1.47)	0.47	0.12
	Weighted median	0.97	(0.83-1.13)	0.67	NA
**Pancreatic**					
**Overall**	Inverse-variance weighted	1.10	(0.91-1.34)	0.32	0.01
	Inverse-variance weighted (random effects)	1.10	(0.84-1.45)	0.48	0.01
	Wald (rs17366568)	1.00	(0.73-1.38)	0.99	NA
	MR-Egger	1.02	(0.56-1.87)	0.95	0.75
	Weighted median	1.02	(0.77-1.34)	0.91	NA
**Overall (men)**	Inverse-variance weighted	1.08	(0.82-1.43)	0.57	0.32
	Wald (rs17366568)	1.15	(0.76-1.73)	0.52	NA
	MR-Egger	1.33	(0.67-2.65)	0.38	0.46
	Weighted median	1.12	(0.78-1.62)	0.54	NA
**Overall (women)**	Inverse-variance weighted	1.08	(0.79-1.48)	0.61	0.03
	Inverse-variance weighted (random effects)	1.08	(0.69-1.70)	0.73	0.03
	Wald (rs17366568)	0.83	(0.50-1.39)	0.49	NA
	MR-Egger	1.01	(0.28-3.63)	0.99	0.88
	Weighted median	0.92	(0.59-1.42)	0.70	NA
**Renal cell carcinoma**					
**Overall**	Inverse-variance weighted	0.93	(0.79-1.08)	0.33	0.37
	Wald (rs17366568)	0.98	(0.75-1.30)	0.90	NA
	MR-Egger	0.91	(0.64-1.29)	0.60	0.91
	Weighted median	0.95	(0.76-1.18)	0.64	NA
**Overall (men)**	Inverse-variance weighted	0.95	(0.69-1.30)	0.73	0.21
	Wald (rs17366568)	0.65	(0.39-1.07)	0.09	NA
	MR-Egger	0.55	(0.28-1.11)	0.13	0.12

	Weighted median	0.77	(0.51-1.15)	0.20	NA
**Overall (women)**	Inverse-variance weighted	0.83	(0.56-1.23)	0.36	0.84
	Wald (rs17366568)	1.18	(0.61-2.31)	0.62	NA
	MR-Egger	1.49	(0.59-3.75)	0.42	0.21
	Weighted median	1.00	(0.60-1.67)	0.99	NA
**Ovarian**					
**Overall**	Inverse-variance weighted	1.07	(0.96-1.19)	0.22	0.53
	Wald (rs17366568)	1.03	(0.89-1.19)	0.69	NA
	MR-Egger	0.95	(0.75-1.19)	0.62	0.21
	Weighted median	1.03	(0.91-1.17)	0.63	NA
**Serous**	Inverse-variance weighted	1.07	(0.95-1.21)	0.25	0.94
	Wald (rs17366568)	1.07	(0.91-1.26)	0.44	NA
	MR-Egger	1.03	(0.81-1.32)	0.79	0.73
	Weighted median	1.07	(0.92-1.23)	0.38	NA
**Clear-cell**	Inverse-variance weighted	1.30	(0.94-1.78)	0.11	0.60
	Wald (rs17366568)	1.42	(0.93-2.16)	0.11	NA
	MR-Egger	1.56	(0.75-3.27)	0.19	0.51
	Weighted median	1.41	(0.97-2.05)	0.07	NA
**Endometrioid**	Inverse-variance weighted	1.05	(0.84-1.32)	0.66	0.28
	Wald (rs17366568)	0.91	(0.67-1.24)	0.56	NA
	MR-Egger	0.73	(0.42-1.26)	0.21	0.11
	Weighted median	0.91	(0.69-1.21)	0.53	NA
**Endometrial**					
**Overall**	Inverse-variance weighted	1.02	(0.89-1.17)	0.75	0.02
	Inverse-variance weighted (random effects)	1.02	(0.83-1.26)	0.84	0.02

	Wald (rs17366568)	1.01	(0.80-1.26)	0.97	NA
	MR-Egger	1.02	(0.55-1.86)	0.95	0.98
	Weighted median	1.01	(0.84-1.21)	0.94	NA

OR: odds ratio; CI: confidence intervals; P-value for pleiotropy in MR-Egger regression; P-value for heterogeneity in inverse-variance weighted analysis.

Supplementary Table 3. Mendelian randomization estimates between adiponectin concentration and colorectal cancer risk using summary estimates of adiponectin unadjusted for body mass index.

Cancer type	Methods	Estimates (OR)	95% CI	P-value	P-value for pleiotropy or heterogeneity
**Colorectal**					
**Overall**	Inverse-variance weighted	0.90	(0.84-0.97)	0.01	0.01
	MR-Egger	0.98	(0.79-1.21)	0.84	0.33
	Weighted median	0.92	(0.83-1.02)	0.12	NA
	Multivariable analysis (BMI adjusted)	0.92	(0.84-1.01)	0.10	0.01
**Overall (men)**	Inverse-variance weighted	0.89	(0.80-0.99)	0.04	0.02
	MR-Egger	1.00	(0.69-1.45)	0.99	0.41
	Weighted median	0.95	(0.82-1.10)	0.50	NA
	Multivariable analysis (BMI adjusted)	0.98	(0.86-1.13)	0.80	0.10
**Overall (women)**	Inverse-variance weighted	0.90	(0.81-1.01)	0.07	0.62
	MR-Egger	0.82	(0.64-1.05)	0.11	0.38
	Weighted median	0.89	(0.77-1.03)	0.10	NA
	Multivariable analysis (BMI adjusted)	0.88	(0.77-1.02)	0.08	0.54
**Colon**	Inverse-variance weighted	0.90	(0.82-0.98)	0.02	0.08
	MR-Egger	0.98	(0.78-1.23)	0.84	0.36
	Weighted median	0.93	(0.82-1.06)	0.29	NA
	Multivariable analysis (BMI adjusted)	0.92	(0.82-1.03)	0.16	0.09
**Rectal**	Inverse-variance weighted	0.91	(0.81-1.03)	0.13	0.05

MR-Egger	1.11	(0.83-1.47)	0.46	0.11
Weighted median	0.97	(0.82-1.14)	0.68	NA
Multivariable analysis (BMI adjusted)	0.94	(0.81-1.09)	0.40	0.04

OR: odds ratio; CI: confidence intervals; P-value for pleiotropy in MR-Egger regression; P-value for heterogeneity in inverse-variance weighted analysis; BMI: Body mass index.

Supplementary Table 4. Mendelian randomization estimates between leptin concentration and cancer risk.

Cancer type	Estimates (OR)[a]	95% CI	P-value	P-value for pleiotropy or heterogeneity
**Colorectal**				
**Overall**	0.99	(0.62-1.57)	0.96	0.32
**Overall (rs10487505)**	0.82	(0.45-1.48)	0.51	NA
**Overall (men)**	1.66	(0.77-3.57)	0.20	0.31
**Overall (men-rs10487505)**	1.19	(0.43-3.24)	0.74	NA
**Overall (women)**	0.69	(0.38-1.27)	0.24	0.72
**Overall (women-rs10487505)**	0.64	(0.30-1.35)	0.24	NA
**Colon**	0.82	(0.47-1.43)	0.48	0.26
**Colon (rs10487505)**	0.63	(0.31-1.30)	0.21	NA
**Rectal**	1.66	(0.80-3.44)	0.18	0.72
**Rectal (rs10487505)**	1.49	(0.59-3.79)	0.40	NA
**Pancreatic**				
**Overall**	0.39	(0.11-1.37)	0.14	0.32
**Overall (rs10487505)**	0.24	(0.05-1.17)	0.08	NA
**Overall (men)**	0.43	(0.06-3.18)	0.41	0.18
**Overall (men-rs10487505)**	0.14	(0.01-1.87)	0.14	NA
**Overall (women)**	0.35	(0.06-1.93)	0.23	0.98
**Overall (women-rs10487505)**	0.35	(0.04-2.94)	0.34	NA
**Renal cell carcinoma**				
**Overall**	0.93	(0.35-2.44)	0.88	0.84
**Overall (rs10487505)**	1.00	(0.29-3.43)	0.99	NA

**Overall (men)**	0.55	(0.09-3.53)	0.53	0.95
**Overall (men-rs10487505)**	0.58	(0.05-6.13)	0.65	NA
**Overall (women)**	2.61	(0.25-27.69)	0.43	0.15
**Overall (women-rs10487505)**	0.70	(0.04-13.77)	0.81	NA
**Ovarian**				
**Overall**	1.78	(0.93-3.38)	0.08	0.99
**Overall (rs10487505)**	1.78	(0.81-3.91)	0.15	NA
**Serous**	1.80	(0.86-3.78)	0.12	0.98
**Serous (rs10487505)**	1.79	(0.72-4.44)	0.21	NA
**Clear-cell**	6.27	(0.90-43.67)	0.06	0.60
**Clear-cell (rs10487505)**	4.33	(0.40-46.87)	0.23	NA
**Endometrioid**	1.13	(0.28-4.54)	0.87	0.42
**Endometrioid (rs10487505)**	1.69	(0.31-9.28)	0.55	NA
**Endometrial**				
**Overall**	1.46	(0.69-3.06)	0.32	0.80
**Overall (rs10487505)**	1.36	(0.55-3.39)	0.50	NA

[a] Effect estimates calculated with the Inverse-variance weighted method. The MR-Egger and Weighted median method cannot be applied for leptin due to the low number of variants (i.e. two variants).

OR: odds ratio; CI: confidence intervals; P-value for pleiotropy in MR-Egger regression; P-value for heterogeneity in inverse-variance weighted analysis.

Supplementary Table 5. Mendelian randomization estimates between soluble leptin receptor concentration and cancer risk.

Cancer type	Methods	Estimates (OR)	95% CI	P-value	P-value for pleiotropy or heterogeneity
**Colorectal**					
**Overall**	Inverse-variance weighted	0.99	(0.98-1.00)	0.20	0.68
	MR-Egger	0.99	(0.95-1.04)	0.57	0.79
	Weighted median	0.99	(0.98-1.01)	0.22	NA
**Overall (men)**	Inverse-variance weighted	1.00	(0.98-1.02)	0.93	0.75
	MR-Egger	1.01	(0.96-1.06)	0.72	0.61
	Weighted median	1.00	(0.98-1.02)	0.92	NA
**Overall (women)**	Inverse-variance weighted	0.98	(0.96-1.00)	0.06	0.78
	MR-Egger	0.98	(0.93-1.03)	0.23	0.90
	Weighted median	0.98	(0.96-1.00)	0.07	NA
**Colon**	Inverse-variance weighted	0.99	(0.98-1.01)	0.35	0.93
	MR-Egger	1.00	(0.97-1.02)	0.76	0.75
	Weighted median	0.99	(0.98-1.01)	0.47	NA
**Rectal**	Inverse-variance weighted	0.98	(0.96-1.00)	0.07	0.26
	MR-Egger	0.98	(0.88-1.10)	0.57	0.91
	Weighted median	0.98	(0.96-1.00)	0.09	NA
**Pancreatic**					
**Overall**	Inverse-variance weighted	1.03	(0.99-1.07)	0.12	0.10
	MR-Egger	1.01	(0.80-1.29)	0.82	0.75
	Weighted median	1.02	(0.98-1.07)	0.26	NA
**Overall (men)**	Inverse-variance weighted	1.06	(1.01-1.12)	0.02	0.08

	MR-Egger	1.03	(0.75-1.41)	0.73	0.63
	Weighted median	1.05	(1.00-1.11)	0.07	NA
**Overall (women)**	Inverse-variance weighted	0.99	(0.94-1.05)	0.77	0.16
	MR-Egger	1.00	(0.71-1.39)	0.98	0.93
	Weighted median	1.00	(0.94-1.06)	0.96	NA
**Renal cell carcinoma**					
**Overall**	Inverse-variance weighted	1.02	(0.99-1.06)	0.12	0.96
	MR-Egger	1.02	(0.92-1.14)	0.44	0.99
	Weighted median	1.02	(0.99-1.06)	0.12	NA
**Overall (men)**	Inverse-variance weighted	1.02	(0.96-1.08)	0.60	0.50
	MR-Egger	1.02	(0.83-1.25)	0.75	0.97
	Weighted median	1.02	(0.96-1.08)	0.61	NA
**Overall (women)**	Inverse-variance weighted	1.04	(0.97-1.12)	0.29	0.07
	MR-Egger	1.05	(0.80-1.36)	0.54	0.91
	Weighted median	1.05	(0.97-1.14)	0.21	NA
**Ovarian**					
**Overall**	Inverse-variance weighted	1.01	(0.99-1.03)	0.35	0.84
	MR-Egger	1.01	(0.96-1.06)	0.68	0.84
	Weighted median	1.01	(0.99-1.03)	0.40	NA
**Serous**	Inverse-variance weighted	1.02	(1.00-1.05)	0.10	0.89
	MR-Egger	1.02	(0.97-1.06)	0.48	0.72
	Weighted median	1.02	(0.99-1.05)	0.16	NA
**Clear-cell**	Inverse-variance weighted	0.99	(0.93-1.06)	0.86	0.09
	MR-Egger	0.96	(0.64-1.45)	0.72	0.70
	Weighted median	0.98	(0.92-1.05)	0.62	NA
**Endometrioid**	Inverse-variance weighted	0.98	(0.94-1.03)	0.52	0.65

	MR-Egger	1.02	(0.92-1.12)	0.69	0.32
	Weighted median	0.99	(0.94-1.04)	0.68	NA
**Endometrial**					
**Overall**	Inverse-variance weighted	1.02	(1.00-1.05)	0.09	0.38
	MR-Egger	1.01	(0.92-1.10)	0.83	0.44
	Weighted median	1.02	(0.99-1.04)	0.22	NA

OR: odds ratio; CI: confidence intervals; P-value for pleiotropy in MR-Egger regression; P-value for heterogeneity in inverse-variance weighted analysis.

Supplementary Table 6. Mendelian randomization estimates between plasminogen activator inhibitor-1 concentration and cancer risk.

Cancer type	Methods	Estimates (OR)	95% CI	P-value	P-value for pleiotropy or heterogeneity
**Colorectal**					
**Overall**	Inverse-variance weighted	1.01	(0.86-1.19)	0.92	0.89
	Wald (rs2227631)	1.04	(0.82-1.33)	0.72	NA
	MR-Egger	1.33	(0.60-2.93)	0.48	0.49
	Weighted median	1.02	(0.85-1.24)	0.81	NA
**Overall (men)**	Inverse-variance weighted	0.94	(0.75-1.19)	0.62	0.97
	Wald (rs2227631)	0.95	(0.68-1.33)	0.78	NA
	MR-Egger	1.15	(0.38-3.48)	0.81	0.72
	Weighted median	0.94	(0.72-1.23)	0.66	NA
**Overall (women)**	Inverse-variance weighted	1.05	(0.83-1.32)	0.71	0.95
	Wald (rs2227631)	1.11	(0.79-1.56)	0.56	NA
	MR-Egger	1.45	(0.46-4.52)	0.52	0.57
	Weighted median	1.08	(0.82-1.42)	0.57	NA
**Colon**	Inverse-variance weighted	0.90	(0.74-1.10)	0.30	0.80
	Wald (rs2227631)	0.96	(0.72-1.29)	0.80	NA
	MR-Egger	1.22	(0.39-3.85)	0.69	0.53
	Weighted median	0.95	(0.75-1.19)	0.64	NA
**Rectal**	Inverse-variance weighted	1.18	(0.91-1.52)	0.21	0.96
	Wald (rs2227631)	1.21	(0.83-1.76)	0.32	NA
	MR-Egger	1.56	(0.45-5.41)	0.48	0.65
	Weighted median	1.18	(0.87-1.58)	0.28	NA

**Pancreatic**					
**Overall**	Inverse-variance weighted	1.13	(0.73-1.76)	0.58	0.70
	Wald (rs2227631)	1.14	(0.60-2.18)	0.68	NA
	MR-Egger	2.28	(0.08-63.81)	0.45	0.51
	Weighted median	1.15	(0.69-1.94)	0.59	NA
**Overall (men)**	Inverse-variance weighted	0.93	(0.52-1.69)	0.82	0.35
	Wald (rs2227631)	1.11	(0.46-2.67)	0.81	NA
	MR-Egger	7.55	(0.06-983.95)	0.22	0.20
	Weighted median	0.90	(0.44-1.86)	0.77	NA
**Overall (women)**	Inverse-variance weighted	1.43	(0.74-2.76)	0.29	0.51
	Wald (rs2227631)	1.17	(0.45-3.09)	0.75	NA
	MR-Egger	0.55	(0.00-558.04)	0.74	0.60
	Weighted median	1.20	(0.54-2.64)	0.66	NA
**Renal cell carcinoma**					
**Overall**	Inverse-variance weighted	1.09	(0.78-1.52)	0.62	0.13
	Wald (rs2227631)	0.72	(0.44-1.18)	0.20	NA
	MR-Egger	0.26	(0.01-9.04)	0.24	0.22
	Weighted median	1.16	(0.73-1.83)	0.54	NA
**Overall (men)**	Inverse-variance weighted	1.16	(0.62-2.19)	0.64	0.25
	Wald (rs2227631)	0.65	(0.26-1.64)	0.36	NA
	MR-Egger	0.08	(0.00-69.67)	0.25	0.22
	Weighted median	1.25	(0.58-2.73)	0.58	NA
**Overall (women)**	Inverse-variance weighted	0.79	(0.36-1.77)	0.57	0.26
	Wald (rs2227631)	0.63	(0.20-2.05)	0.45	NA
	MR-Egger	2.23	(0.00-11240.93)	0.72	0.65
	Weighted median	0.80	(0.31-2.11)	0.67	NA

**Ovarian**					
**Overall**	Inverse-variance weighted	1.22	(0.95-1.55)	0.11	0.53
	Wald (rs2227631)	1.36	(0.95-1.94)	0.10	NA
	MR-Egger	1.65	(0.12-22.04)	0.50	0.66
	Weighted median	1.26	(0.94-1.70)	0.12	NA
**Serous**	Inverse-variance weighted	1.16	(0.87-1.53)	0.31	0.26
	Wald (rs2227631)	1.50	(0.99-2.27)	0.05	NA
	MR-Egger	2.20	(0.05-98.65)	0.47	0.54
	Weighted median	1.31	(0.92-1.88)	0.14	NA
**Clear-cell**	Inverse-variance weighted	1.02	(0.48-2.13)	0.97	0.15
	Wald (rs2227631)	0.41	(0.14-1.23)	0.11	NA
	MR-Egger	0.02	(0.00-6.32)	0.10	0.10
	Weighted median	1.27	(0.47-3.40)	0.64	NA
**Endometrioid**	Inverse-variance weighted	1.94	(1.14-3.31)	0.01	0.62
	Wald (rs2227631)	1.63	(0.75-3.56)	0.22	NA
	MR-Egger	2.18	(0.01-461.82)	0.60	0.93
	Weighted median	1.86	(0.98-3.52)	0.06	NA
**Endometrial**					
**Overall**	Inverse-variance weighted	1.38	(1.04-1.82)	0.03	0.08
	Wald (rs2227631)	1.12	(0.74-1.70)	0.59	NA
	MR-Egger	1.65	(0.01-406.40)	0.73	0.90
	Weighted median	1.18	(0.83-1.68)	0.35	NA

OR: odds ratio; CI: confidence intervals; P-value for pleiotropy in MR-Egger regression; P-value for heterogeneity in inverse-variance weighted analysis.

Supplementary Table 7. Associations with potential confounders of the selected variants for adipokines.

SNP	Associated with potential confounders	Associated with obesity-related confounders	Adipokine
rs2791552	Adiposity, Arm fat mass left, Arm fat mass right, Arm fat percentage left, Arm fat percentage right, Body fat percentage, Fasting insulin, Hip circumference, Impedance of arm left, Impedance of arm right, Impedance of whole body, Leg fat mass left, Leg fat mass right, Leg fat percentage left, Leg fat percentage right, log Fasting insulin, Trunk fat mass, Trunk fat percentage, Waist circumference adjusted for smoking in females, Waist hip ratio, Whole body fat mass	Yes	adiponectin
rs2943641	Adiposity, Appendicular lean mass, Arm fat percentage left, Arm fat percentage right, Arm fat-free mass left, Arm fat-free mass right, Arm predicted mass left, Arm predicted mass right, Body fat percentage, Body mass index, Diabetes diagnosed by doctor, Fasting insulin, HDL cholesterol, Hypertension, Illnesses of mother: diabetes, Impedance of arm left, Impedance of arm right,	Yes	adiponectin

	Impedance of whole body, Insulin sensitivity index adjusted for BMI, IRS1 gene expression in human omental fat, IRS1 gene expression in Omental fat tissue from patients who underwent bariatric surgery, IRS1 gene expression in Subcutaneous fat tissue from the general population, IRS1 gene expression in Subcutaneous fat tissue from the general population male, IRS1 Trancript NM005544 expression in Adipose tissue, Lean body mass, log Fasting insulin, Medication for cholesterol, blood pressure or diabetes: blood pressure medication, Percent body fat, Self-reported diabetes, Self-reported high cholesterol, Treatment with metformin, Triglycerides, Trunk fat mass, Trunk fat percentage, Trunk fat-free mass, Trunk predicted mass, Type 2 diabetes, Waist circumeference in physically active males, Waist circumference in males, Waist circumference in physically active females, Waist hip ratio in males		
rs2276853	Granulocyte count, Lymphocyte count, Myeloid white cell count, Platelet distribution width, Sum neutrophil eosinophil counts, White blood cell count	No	adiponectin

rs3087866	Height, High light scatter percentage of red cells, High light scatter reticulocyte count, Impedance of arm left, Impedance of arm right, Impedance of whole body, Mean corpuscular hemoglobin, Mean corpuscular volume, Pulse rate, Reticulocyte count, Reticulocyte fraction of red cells, Sitting height	No	adiponectin
rs13303	Age at menarche, Cross disorder, Hematocrit, Hemoglobin concentration, High light scatter percentage of red cells, High light scatter reticulocyte count, Impedance of arm left, Impedance of arm right, Impedance of leg left, Impedance of leg right, Impedance of whole body, Intelligence multi trait analysis, NT5DC2 gene expression in human adipocytes, Red blood cell count, Reticulocyte count, Reticulocyte fraction of red cells, Schizophrenia, Waist circumference adjusted for BMI, Waist hip ratio adjusted for BMI, Worrier or anxious feelings	Yes	adiponectin
rs17366568	-	-	adiponectin
rs13133548	Arm fat percentage left, Arm fat percentage right, Body fat percentage, Body mass index, Fasting insulin, HDL cholesterol, High density lipoprotein,	Yes	adiponectin

rs2925979	Hip circumference, Impedance of arm left, Impedance of arm right, log Fasting insulin adjusted for BMI, Trunk fat mass, Trunk fat percentage, Waist circumference adjusted for smoking in females, Waist to hip ratio adjusted for body mass index, Whole body fat mass Cholesterol hdl, HDL cholesterol, High density lipoprotein, Impedance of arm left, Impedance of arm right, Self-reported hypertension, Treatment with cholesterol lowering medication, Type 2 diabetes, Vascular or heart problems diagnosed by doctor: high blood pressure, Vascular or heart problems diagnosed by doctor: none of the above, Waist hip ratio	Yes	adiponectin
rs10282707	Arm fat-free mass left, Arm fat-free mass right, Arm predicted mass left, Arm predicted mass right, HDL cholesterol, High density lipoprotein, Trunk fat-free mass, Trunk predicted mass, Whole body fat-free mass, Whole body water mass	Yes	adiponectin
rs3735080	Lymphocyte count, Lymphocyte percentage of white cells, Neutrophil percentage of white cells, Impedance of leg left	No	adiponectin
rs7134375	Cholesterol hdl, Pulse rate	No	adiponectin
rs10861661	Comparative height size at age 10,	No	adiponectin

	Height, Sitting height		
rs11057405	Body mass index, Hip circumference, High density lipoprotein, Arm fat mass left, Arm fat mass right, Arm fat percentage left, Arm fat percentage right, Arm fat-free mass left, Arm fat-free mass right, Arm predicted mass left, Arm predicted mass right, Basal metabolic rate, Body fat percentage, Height, Leg fat mass left, Leg fat mass right, Leg fat percentage left, Leg fat percentage right, Leg fat-free mass left, Leg fat-free mass right, Leg predicted mass left, Leg predicted mass right, Trunk fat mass, Trunk fat percentage, Trunk fat-free mass, Trunk predicted mass, Waist circumference, Weight, Whole body fat mass, Whole body fat-free mass, Whole body water mass	Yes	adiponectin
rs11057353	-	-	adiponectin
rs4311394	Cholesterol hdl, HDL cholesterol, High density lipoprotein, Fasting insulin adjusted for BMI, Reticulocyte count, Reticulocyte fraction of red cells	Yes	adiponectin
rs3865188	-	-	adiponectin
rs145119400	-	-	adiponectin
rs4805885	Arm fat percentage left, Arm fat percentage right, Body fat percentage, High density lipoprotein, Impedance of	Yes	adiponectin

	arm left, Impedance of arm right, Impedance of whole body, log Fasting insulin adjusted for BMI, PEPD gene expression in human adipocytes, Trunk fat mass, Trunk fat percentage, Whole body fat mass		
rs10487505	Comparative body size at age 10	Yes	leptin
rs6071166	-	-	leptin
rs17415296	Blood protein levels, White blood cell count	No	leptin receptor
rs4655537	Baseline acute phase serum amyloid concentrations, Blood protein levels, C reactive protein, Granulocyte count, Granulocyte percentage of myeloid white cells, Mean corpuscular hemoglobin, Myeloid white cell count, Neutrophil count, Plasma C reactive protein female, Sum basophil neutrophil counts, Sum neutrophil eosinophil counts, White blood cell count	No	leptin receptor
rs17412403	Granulocyte count, Granulocyte percentage of myeloid white cells, Myeloid white cell count, Neutrophil count, Sum basophil neutrophil counts, Sum neutrophil eosinophil counts, Blood protein levels, Age at menarche, Comparative body size at age 10, Impedance of arm, Impedance of whole body, White blood cell count	Yes	leptin receptor

rs7535099	Blood protein levels	No	leptin receptor
rs11128603	Type 2 diabetes, Arm fat mass, Arm fat percentage, Body fat percentage, Impedance of arm, Impedance of leg, Impedance of whole body, Leg fat mass, Leg fat percentage, Trunk fat mass, Trunk fat percentage, Whole body fat mass, Waist circumference, Fasting insulin, Body mass index, Proximal gene expression in macrophages PPARG gene	Yes	plasminogen activator inhibitor-1
rs2227631	-	-	plasminogen activator inhibitor-1
rs6976053	Heart rate, Proximal gene expression in monocytes II TRIP6 gene, Proximal gene expression in monocytes II UFSP1 gene, Proximal gene expression in monocytes TRIP6 gene, Proximal gene expression in monocytes UFSP1 gene, Proximal gene expression in the liver MUC3A gene, Pulse rate, RR interval	No	plasminogen activator inhibitor-1
rs6486122	Age at menarche, Body mass index	Yes	plasminogen activator inhibitor-1
